# Reliability and validity of Arabic version of Lower Urinary Tract Dysfunction Research Network Symptom Index-10 questionnaire (LURN SI-10)

**DOI:** 10.1007/s00345-026-06210-w

**Published:** 2026-02-16

**Authors:** Fady K. Ghobrial, Ali Ibrahim, Moaaz Younes, Abdelkarim Alrubat, Salem Bahdilh, Mohamed Abd Elbaset, Diaa-Eldin Taha

**Affiliations:** 1 Urology Department, Faculty of Medicine, Damietta university, New Damietta City, Egypt; 2 Urology Department, Faculty of Medicine, Kafrelsheikh university, Kafr el-Sheikh, Egypt; 3 Urology Department, King Saud Medical City, Riyadh, Saudi Arabia; 4 Urology Department, Faculty of Medicine, Horus University, New Damietta City, Egypt

**Keywords:** LUTs, LURN, LURN SI-10, IPSS, Arabic validation

## Abstract

**Purpose:**

To evaluate the validity and reliability of the Arabic version of the Lower Urinary Tract Dysfunction Research Network Symptom Index-10 (LURN SI-10) questionnaire for assessing lower urinary tract symptoms (LUTs) in Arabic-speaking patients.

**Methods:**

The LURN SI-10 was translated into Arabic following the Patient-Reported Outcome Consortium guidelines, with back-translation and a pilot study among 15 patients for clarity. The Arabic version of LURN-SI 10, and the validated Arabic versions of International Prostate Symptom Score (IPSS) and International Consultation on Incontinence Questionnaire Overactive Bladder (ICIQ-OAB) questionnaires were administered for patients with LUTs. Reliability, internal consistency and concurrent validity of LURN-SI 10 were established.

**Results:**

From October 2024 to February 2025, 186 patients with LUTS were enrolled. The Arabic LURN SI-10 demonstrated high reliability (Cronbach’s alpha = 0.8; all subdomains > 0.7). Significant positive correlations were found between LURN SI-10 with IPSS (*r* = 0.780, 0.833, 0.683, *p* < 0.001) and ICIQ-OAB (*r* = 0.730, 0.768, 0.674 *p* < 0.001) for all patients, males and females respectively. Frequency, urgency, nocturia, and weak stream domains of LURN SI-10 correlated significantly with their IPSS counterparts. Frequency, nocturia, urgency, and urge urinary incontinence (UUI) correlated with ICIQ-OAB domains. Significant positive correlations were noted between pain, urgency, and other domains, including post-void dribbling (PVD), bladder pain, and bother.

**Conclusion:**

The Arabic LURN SI-10 is a valid and reliable tool for assessing LUTs, showing strong correlations with IPSS and ICIQ-OAB. The comprehensive assessment of PVD, UUI, stress urinary incontinence, and bladder pain enhances its utility over IPSS for both male and female patients with LUTS.

## Introduction

Lower urinary tract symptoms (LUTs) have a negative impact on health-related quality of life (QoL), general and mental health status [1]. LUTs in men are frequently assessed by international prostate symptom score (IPSS) as it was developed for them [2]. However, LUTs are divided into storage, voiding and postmicturition symptoms [3]. IPSS does not entail questions for assessment of urinary incontinence [2]. Moreover, it lacks the assessment of post-micturition symptoms and bother caused by each separate symptom [4].

On the other hand, The International Consultation on Incontinence Questionnaire Overactive Bladder (ICIQ-OAB) is a questionnaire for evaluating overactive bladder and related impact QoL and outcome of treatment in men and women. It provides a measure to assess the impact of urinary frequency, urgency, urge incontinence (UI) and nocturia symptoms with no domains for voiding LUTs [5].

To overcome these shortcomings, Lower Urinary Tract Dysfunction Research Network (LURN) investigators developed The Symptoms of Lower Urinary Tract Dysfunction Research Network Symptom Index-29 (LURN SI-29) questionnaire to improve the research and clinical outcome measurements for both men and women with LUTS [6]. For more practical daily use, they reduced the LURN SI-29 to a brief and comprehensive form covering the major LUTs in men and women which is LURN SI-10. It assess urinary urgency, incontinence, bladder pain voiding and post-micturition symptoms, frequency, nocturia [7].

European association of urology (EAU) guidelines 2024 recommend the use of LURN SI-10 questionnaire as it correlates strongly with the IPSS [8]. Furthermore, It provides additional imperative information about the incontinence status and bladder pain in patients with LUTs [9].

To our knowledge, there is no study aimed at the external validity of LURN SI-10 for Arabic language. In this study, we aim to evaluate the validity and reliability of the Arabic version of LURN SI-10.

## Materials and methods

Official permission from the LURN team to translate LURN SI-10 into Arabic was obtained. The study was conducted after the approval of local ethical committee (code: MKSU20-2–24). Patients with LUTs who were referred to urology outpatient clinics of two tertiary centers were enrolled in the study. The Inclusion criteria for participants were: age over 18 years, answering all questionnaires’ questions and signing an informed consent. Patients who were unable to communicate, illiterate or had cognitive disorders were excluded.

### Tools for data collection

Patients’ demographic data as age, sex, body mass index and medical history were collected. The participants were asked to answer questions of the Arabic version of LURN-SI 10, and the validated Arabic versions of IPSS and ICIQ-OAB questionnaires on their own without professional aid.

### LURN SI-10

The LURN SI-10 is a short-form clinical assessment derived from the longer LURN Comprehensive Assessment of Self-Reported Urinary Symptoms (CASUS) developed in 2019. It includes 10 domains which are urinary urgency, urge urinary incontinence (UUI), stress urinary incontinence (SUI), incontinence with exercise, bladder pain, delay, voiding symptoms, post-micturition symptoms, frequency and nocturia with score range 0 to 38, and one question to assess the urinary bothering symptoms [7].

### IPSS

In 1993, the World Health Organization adopted the IPSS, which formally added a bother score to the established 7 items, evaluating the bladder incomplete emptying, frequency, intermittency, urgency, weak stream, straining, and nocturia, and one question about the overall QoL. The total score ranges 0–35 [10].

### ICIQ-OAB

It is at tool to evaluate the symptoms of OAB and the associated bothering. It involves urinary frequency (ICIQ-OAB-3), nocturia (ICIQ-OAB-4), urgency (ICIQ-OAB-5), and urge incontinence (ICIQ-OAB-6) [5].

### Linguistic and content validation

The translation of LURN SI-10 questionnaire from English to Arabic were carried out based on guidelines Patient-Reported Outcome (PRO) Consortium translation process provided by Eremenco et al. [11]. Two bilingual translators independently, whose native language is Arabic translated the original version of the questionnaires from English into Arabic language. Afterward, the initial Arabic forms were back translated to the original language (English) by an English native speaker, fluent in Arabic with no medical background and blind to the study purpose. The authors were convened to review the English back-translation to reveal any inconsistencies with the Arabic version. The items of the questionnaire were evaluated to determine the clinical relevance, suitability for use in the patients’ population, the semantic, idiomatic, and conceptual equivalence of each version item. The proposed modifications in translated Arabic version were accepted when the percentage of agreement between the group of translation and revision was about 90%.

The last step was a pilot study of the Arabic version of the LURN SI-10 among 15 Arabic patients with LUTS (7 women and 8 men) to evaluate their understanding of the items of the questionnaire and whether the alternatives were clear. Minor adjustments were made by the committee according to the comments of the 15 participants. After adjustments, the final Arabic version was concluded and reviewed by LURN team (available at Symptoms of Lower Urinary Tract Dysfunction Research Network (LURN) > Resources > Questionnaires).

#### Statistical analysis

The mean and standard deviation (SD) or median and interquartile range (IQR) for continuous data, numbers and percentages for categorical data were used when appropriate. Cronbach’s *α* test was utilized to evaluate the internal consistency of LURN SI-10. Values were equal to or higher than 0.7 were interpreted as reliable [12]. The concurrent external validity of LURN SI-10 score with IPSS and ICIQ-OAB scores were assessed by Spearman correlation test. The correlation was weak, moderate, and strong when the correlation coefficients equaled 0.1, 0.3 and 0.5 respectively. *p* value less than 0.05 was considered statistically significant. SPSS IBM^®^ version 21 was used for statistical analysis.

## Results

One hundred eighty-six patients completed the study questionnaires between October 2024 and February 2025. Demographic data of the study participants are displayed in Table 1. Median (IQR) score of LURN SI-10 was 12 (7–17), for IPSS was 15 (9–22) and for ICIQ-OAB was 5 [3–7].

**Table 1:** Demographic characteristics and questionnaire scores of the participants

Age mean (±SD) years	51.1 ± 17.3
Sex	
Male No (%)	98 (52.7)
Female No (%)	88 (47.3)
BMI mean (±SD) kg/m^2^	28.8 ± 5.5
Medical history	
DM No (%)	19 (10.2)
HTN No (%)	11 (5.9)
HTN DM No (%)	13 (7)
MS No (%)	22 (11.8)
IHD No (%)	2 (1.1)
DM, HTN, IHD No (%)	5 (2.7)
LURN SI-10 median (IQR)	
All patients	12 (7-17)
Male	10 (5-17)
Female	13 (9-17)
IPSS median (IQR)	
All patients	15 (9-22)
Male	15 (8-21)
Female	16 (10-23)
ICIQ-OAB median (IQR)	
All patients	5 (3-7)
Male	5 (3-7)
Female	5 (3-7)

*BMI* Body mass index, *DM* Diabetes mellitus, *HTN* Hypertension, *ICIQ*-*OAB* International consultation on incontinence questionnaire overactive bladder, *IHD* Ischemic heart disease *IPSS* International prostate symptom score; *IQR* Interquartile range, *LURN SI-10* The symptoms of lower urinary tract dysfunction research network symptom index-10, *MS* Multiple sclerosis, *SD* Standard deviation

Fur the reliability of the scale, Cronbach’s alpha coefficient of the LURN SI-10 was 0.8 and for all subdomains were greater than 0.7 (Table 2).

**Table 2:** Internal consistency (Cronbach’s α) and interdomain association by spearman’s correlation coefficient

	Cronbach’s α		Urgency	Urge incontinence	Stress incontinence	Incontinence with exercise	Bladder pain	Delay	Slow or weak flow	Postvoiding drippling	Frequency	Nocturia	Bothering
Urgency	0.80	*r* *p*		0.28* < 0.001	0.27* < 0.001	0.22* 0.003	0.49* < 0.001	0.26* < 0.001	0.3* < 0.001	0.3* < 0.001	0.23* 0.001	0.22* 0.003	0.45* < 0.001
Urge incontinence	0.80	*r* *p*	0.28* < 0.001		0.57* < 0.001	0.52* < 0.001	0.29* < 0.001	0.04 0.63	0.07 0.34	0.42* < 0.001	0.1 0.16	0.1 0.17	0.37* < 0.001
Stress incontinence	0.80	*r* *p*	0.27* < 0.001	0.57* <0.001		0.69* < 0.001	0.33* < 0.001	0.09 0.23	0.19* 0.01	0.5* < 0.001	0.12 0.11	0.12 0.09	0.35* < 0.001
Incontinence with exercise	0.80	*r* *p*	0.22* 0.003	0.52* <0.001	0.69* < 0.001		0.25* 0.001	0.12 0.09	0.11 0.13	0.44* < 0.001	0.12 0.11	0.12 0.11	0.31* < 0.001
Bladder pain	0.78	*r* *p*	0.49* < 0.001	0.29* < 0.001	0.33* < 0.001	0.25* 0.001		0.43* < 0.001	0.37* < 0.001	0.42* < 0.001	0.29* < 0.001	0.22* 0.002	0.56* < 0.001
Delay	0.81	*r* *p*	0.26* < 0.001	0.04 0.63	0.09 0.23	0.12 0.09	0.43* < 0.001		0.54* < 0.001	0.27* < 0.001	0.17* 0.02	0.1 0.17	0.4* < 0.001
Slow or weak flow	0.81	*r* *p*	0.3* < 0.001	0.07 0.34	0.29* 0.01	0.11 0.13	0.37* < 0.001	0.54* < 0.001		0.32* < 0.001	0.17* 0.02	0.09 0.23	0.47* < 0.001
Postvoiding drippling	0.79	*r* *p*	0.3* < 0.001	0.42* < 0.001	0.5* < 0.001	0.44* < 0.001	0.42* < 0.001	0.27* < 0.001	0.32* < 0.001		0.19* 0.008	0.08 0.28	0.48* < 0.001
Frequency	0.81	*r* *p*	0.23* 0.001	0.1 0.16	0.12 0.11	0.12 0.11	0.29* < 0.001	0.17* 0.02	0.17* 0.02	0.19* 0.008		0.27* < 0.001	0.3* < 0.001
Nocturia	0.82	*r* *p*	0.22* 0.003	0.1 0.17	0.12 0.1	0.12 0.11	0.22* < 0.001	0.1 0.17	0.09 0.23	0.08 0.28	0.27* < 0.001		0.23* 0.002
Bothering	0.78	*r* *p*	0.45* < 0.001	0.37* < 0.001	0.35* < 0.001	0.31* < 0.001	0.56* < 0.001	0.4* < 0.001	0.47* < 0.001	0.48* < 0.001	0.3* < 0.001	0.23* 0.002	

*p *p value,* r *Spearman’s correlation coefficient

*Significant correlation

For internal consistency, a significant low to moderate positive correlation was detected between bladder pain with different urgency domains (r from 0.25 to less than 0.5, *p* < 0.001). Significant positive correlations were noted between delay, weak stream, post voiding drippling (PVD), frequency, urgency, bladder pain and bothering domains, most of these correlations were low to moderate in degree. SUI, UUI, urinary incontinence with exercise exhibited significant strong correlations with each other (*r* > 0.5, *p* < 0.001). PVD domain shows significant low to moderate positive correlations with all domains except nocturia (Table 2).

**Fig. 1:**
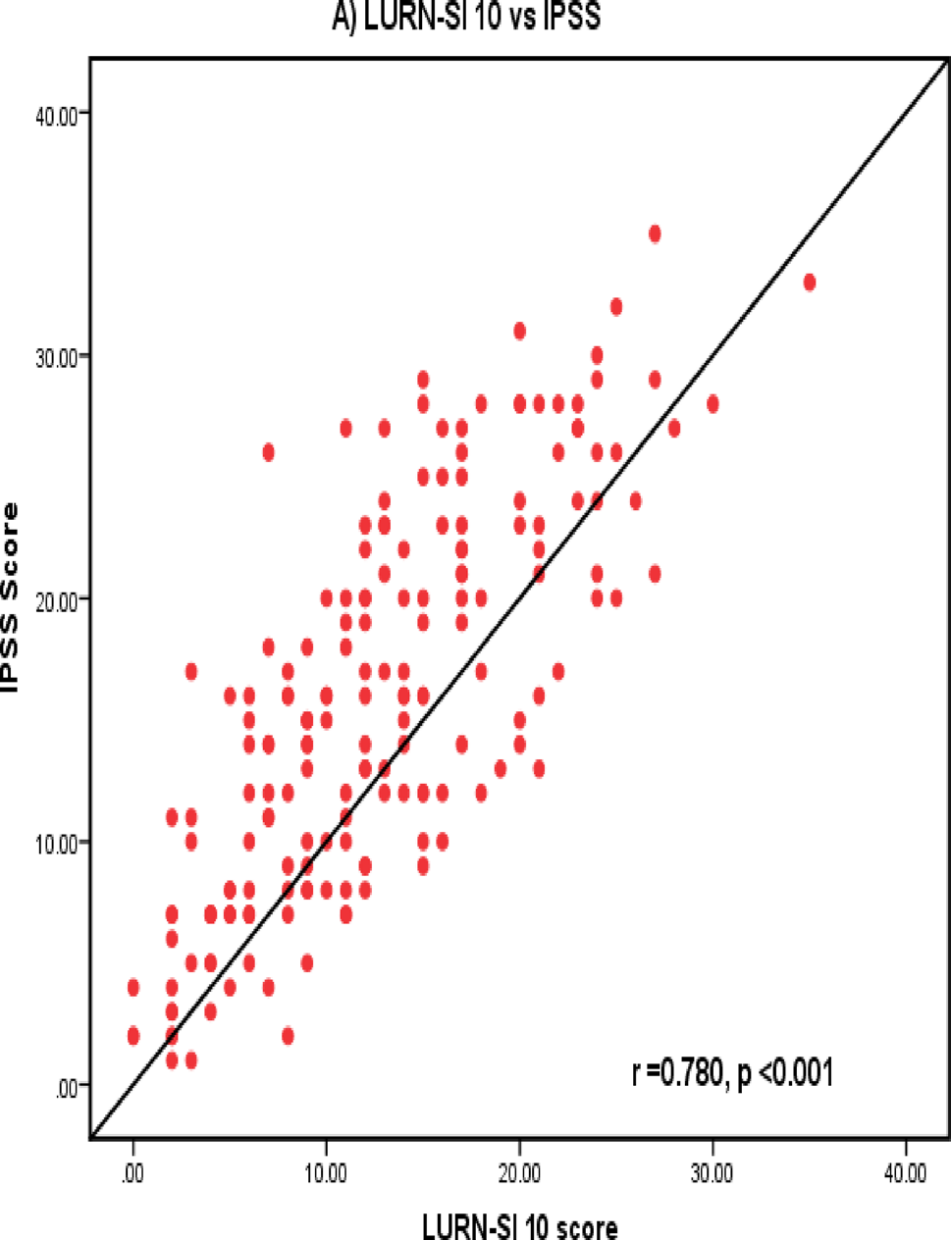

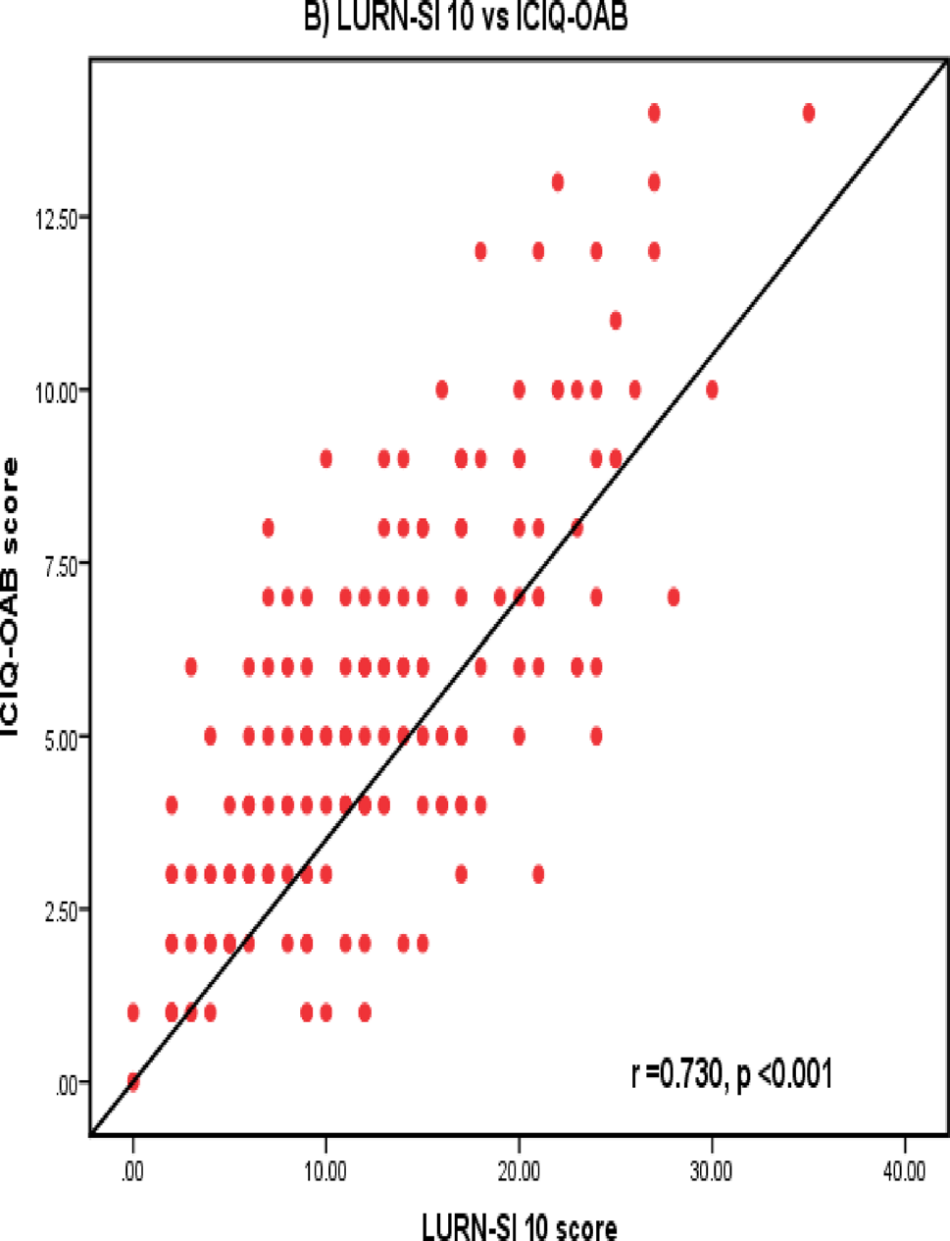
**A** Correlation between LURN SI-10 and IPSS, **B** Correlation between LURN SI-10 and ICIQ-OAB

LURN SI-10 showed a significant positive strong correlation with IPSS and ICIQ-OAB total scores (*r* = 0.780, *p* < 0.001 and *r* = 0.730, *p* < 0.001, respectively) (Fig. 1). In addition, there were significant positive correlations between LURN-10 and both IPSS and ICIQ-OAB in males and females (Table 3). Significant positive correlation was detected between frequency, urgency, slow or weak stream, nocturia domains of LURN SI-10 with their counterpart domains of IPSS questionnaire. Additionally, frequency, nocturia, urgency and UUI were significantly correlated with ICIQ-OAB domains (Table 4).

**Table 3:** The correlations of LURN-10 with IPSS and ICIQ-OAB in males and females

		IPSS score	ICIQ-OAB score
Male LURN SI-10 score	r	0.833*	0.768*
	p	< 0.001	< 0.001
Female LURN SI-10 score	r	0.683*	0.674*
	p	< 0.001	< 0.001

*ICIQ-OAB *International consultation on incontinence questionnaire overactive bladder,* IPSS *International prostate symptom score,* LURN SI-10* The symptoms of lower urinary tract dysfunction research network symptom index-10,* p *p value,* r *Spearman’s correlation coefficient

*significant correlation

**Table 4:** Correlation between LURN SI-10, IPSS and ICIQ-OAB items

LURN SI-10	IPSS			ICIQ-OAB		
	IPSS question No.	*r*	*p*	ICIQ-OAB question No.	*r*	*p*
Q1 Urgency	4	0.52	< 0.001	5a	0.53	< 0.001
Q2 Urge incontinence				6a	0.54	< 0.001
Q3 Stress incontinence						
Q4 Incontinence with exercise						
Q5 Pain						
Q6 Delay						
Q7 Slow or weak flow	5	0.89	< 0.001			
Q8 Postvoiding drippling						
Q9 Frequency	2	0.59	< 0.001	3a	0.9	< 0.001
Q10 Nocturia	7	0.81	< 0.001	4a	1	< 0.001
Bothering	QoL question	0.76	< 0.001			

*ICIQ-OAB* International consultation on incontinence questionnaire overactive bladder,*IPSS* International prostate symptom score,*LURN SI-10* The symptoms of lower urinary tract dysfunction research network symptom index-10,*p*p value,* QoL*Quality of life,*r* Spearman’s correlation coefficient

*significant correlation

## Discussion

IPSS is the most commonly used questionnaire for assessment of male LUTs [13]. Although it was developed for men, some studies have utilized it for women [14]. Unlike IPSS and ICIQ- OAB, LUTs and other urinary symptoms of men and women were taken into consideration during the development of LURN SI-10 questionnaire. It identifies PVD, SUI, UUI and bladder pain/discomfort, which are not depicted with the IPSS. These additional symptomatology assessments improve the evaluation and treatment of patients with LUTs [9]. Our study aimed to translate LURN-SI 10 into Arabic and provides evidence of the content validity of this questionnaire for both males and females suffering from LUTs.

The Arabic version of LURN-SI 10 and its subdomains depicted high reliability and internal consistency (Table 2). The Cronbach’s alpha coefficient of the total scale was 0.8 which is similar to the Turkish version of the questionnaire [15]. Moreover, the subdomains Cronbach’s alpha coefficients were greater than 0.7 (Table 2).

Most of the subdomains showed significant positive correlation with each other (Table 2). However, most of these correlations were of low to moderate degree, they are still statistically significant. Significant positive correlations were detected between storage and voiding symptoms domains with the other domains -which are not included within IPSS- as PVD, bladder pain and bothering. So, LURN-SI 10 provide superior comprehensive tool for patients with LUTS. The incontinence domains did not correlate with delay, frequency and nocturia domains while they correlated with PVD, bladder pain and bothering. This pattern suggests that incontinence symptoms may share underlying distinct pathophysiological pathways or clinical associations with pain and discomfort-related domains, rather than frequency-related symptoms.

The concurrent validity of LURN-SI 10 was confirmed by the statistically significant positive correlation between LURN-SI 10 with IPSS and ICIQ-OAB scores (*r* = 0.780, 0.730 respectively) (*p* < 0.001) (Fig. 1). Other studies found similar statistically significant positive correlation between LURN-SI 10 with IPSS scores (*r* = 0.70 to 0.82, *p* < 0.001) [7, 9, 15]. Strong significant positive correlations were observed in males and females between LURN-SI 10 with IPSS and ICIQ-OAB scores. Cella et al. and detected significant positive correlation between LURN-SI 10 with IPSS in both males and females [7]. Moreover, Akan et al. observed significant positive correlation between LURN-SI 10 with IPSS and OAB-V8 score in both males and females [15]. These results ensured the strength of LURN-SI 10 in evaluating patients’ LUTs regardless their sex. Furthermore, we detected statistically significant positive correlation between four domains of LURN-SI 10 with their equivalent domains of IPSS. Similarly, ICIQ-OAB domains showed statistically significant positive correlation with the same domains in LURN-SI 10. These prove the external validity of LURN SI-10 with other commonly used questionnaires.

The study demonstrates several strengths. The Arabic LURN SI-10 was developed through a rigorous translation process following the PRO Consortium guidelines, incorporating forward and back-translation, pilot testing, and LURN team review, ensuring linguistic and cultural validity. The questionnaire exhibited high reliability and validity, with strong internal consistency and significant correlations with established tools like IPSS and ICIQ-OAB. Finally, the study’s robust sample size of 186 patients provided sufficient statistical power to evaluate the psychometric properties of the Arabic LURN SI-10.

The study has several limitations. Firstly, it did not assess the test-retest reliability of the Arabic LURN SI-10, which limits understanding of the questionnaire’s consistency over time. Secondly, the study did not evaluate the Arabic LURN SI-10 in patients before and after medical or surgical interventions, restricting its validation for monitoring treatment outcomes. Lastly, the study was conducted in only two tertiary centers, which may not fully represent the broader Arabic-speaking population with LUTS.

## Conclusion

The validity and reliability of the Arabic version of LURN SI-10 questionnaire in patients with LUTS were proven. It correlates well with the IPSS and ICIQ-OAB. Its superiority in illustrating the condition of patients with LUTS is evident owing to the presence of additional domains like PVD, UUI, SUI, and bladder pain, which are not entailed with the IPSS.

## Data Availability

No datasets were generated or analysed during the current study.
